# Female Sexual Dysfunction: A Case Study of Disease Mongering and Activist Resistance

**DOI:** 10.1371/journal.pmed.0030178

**Published:** 2006-04-11

**Authors:** Leonore Tiefer

## Abstract

Tiefer highlights key steps in the "creation" of a new diagnosis, female sexual dysfunction, and of the campaign to challenge its reductionist approach to women's sexual problems.


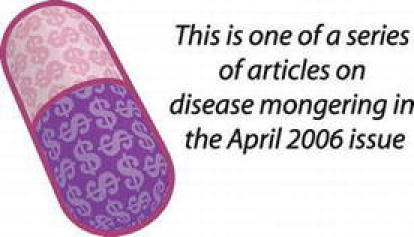


The creation and promotion of “female sexual dysfunction” (FSD) is a textbook case of disease mongering by the pharmaceutical industry and by other agents of medicalization, such as health and science journalists, healthcare professionals, public relations and advertising firms, contract research organizations, and others in the “medicalization industry.” Whether one relies on Lynn Payer's original definition of disease mongering (“trying to convince essentially well people that they are sick, or slightly sick people that they are very ill” [ 1]), her checklist ( Box 1), or the analysis of our pill-popping society that was recently offered by Greg Critser [ 2], the sequence of events and cast of participants involved in FSD matches the classic disease-mongering tactics [ 1, 2].

Each physical condition or life event that has been subject to disease-mongering tactics has its own unique history. Sexual life has become vulnerable to disease mongering for two main reasons. First, a long history of social and political control of sexual expression created reservoirs of shame and ignorance that make it difficult for many people to understand sexual satisfaction or cope with sexual problems in rational ways. Second, popular culture has greatly inflated public expectations about sexual function and the importance of sex to personal and relationship satisfaction.

Thus the public is led to want and expect high rewards from sexual life without having tools to achieve these rewards. People fed a myth that sex is “natural”—that is, a matter of automatic and unlearned biological function—at the same time as they expect high levels of performance and enduring pleasure, are likely to look for simple solutions. This sets the stage for disease mongering, a process that encourages the conversion of socially created anxiety into medical diagnoses suitable for pharmacological treatment.

In this essay, I begin by examining sexual attitudes in the 20th century that were crucial in setting the scene for the creation of FSD. I then highlight key steps in the history of FSD and of the campaign to challenge its reductionist approach to women's sexual problems.

## Setting the Scene: Sex and the 20th Century

In the early 20th century, sexual life and interest were stimulated by intensive urbanization and immigration that disrupted old community-based patterns of sexual regulation [ 3, 4]. Sexual choices and expectations, however, were still largely governed by traditional religion and a double standard. Public discourse around sex was moralistic, and sex-education materials were limited and stigmatized. By mid-century, surveys showed wide variation in sexual habits, with behavior patterns related to social class, gender, cohort, and other background factors [ 5].

Dramatic liberalization occurred after World War II as purity campaigns failed to hold back the sex-promoting impact of changes in longevity, leisure, employment and childrearing, new technology, and mass media [ 4]. Effective oral contraceptives and medical treatments for venereal diseases removed sexual inhibitions due to fear of pregnancy and disease. A youth culture of charged eroticism developed. Changes in obscenity laws permitted sexual explicitness in the mass media. The women's and gay- and lesbian-rights movements of the 1960s and 1970s raised the importance of sexual behavior and identity. Each new technological development in communications—movies, car radios, television, videotapes, Internet—was used to promote sex-related products and to escalate the importance of sexual life and the availability of stimulation.

## Medicalizing Sexuality

Beginning in the 1970s, along with the increasing sexual explicitness in popular culture, there were two competing academic theories of sexuality. In the social sciences and humanities, a social-constructionist perspective emphasized political, economic, and social determinants of sexual life [ 6]. It tended to see learning and education as keys to sexual satisfaction. In psychology and medicine, by contrast, a reductionist view of sexuality prevailed that stressed universal, evolution-based patterns of sexual motive, attraction, and conduct. This view saw satisfaction as an inherent result of normal function. In truth, however, there wasn't much academic sex research of any sort, as the topic was controversial and hence underfunded. There were very few academic or professional training programs, and sexological organizations, conferences, and journals were lively but small and somewhat defensive, rather than parts of an established specialty area of sexuality studies.

In the 1980s, the nature of sex research and expertise began to shift as a new “sexual medicine” focused on function was created by urologists, insurance reimbursement programs, diagnostic technologies, science and medicine journalists, and, then, the pharmaceutical industry [ 7].

Urologists looked to new opportunities in genitourinary sexual medicine as their surgical careers were limited by the new (1984) kidney stone lithotripsy and by effective medications for benign prostate disease. Insurance-based reimbursement for sex-problem treatments (including psychotherapy) became linked to a diagnostic classification system that recognized only discrete sexual “dysfunctions” such as low desire, inadequate arousal/erection, and premature or delayed orgasm/ejaculation. Technologies for measuring genital blood flow and nerve function were widely used to substantiate dysfunction diagnoses. Taking advantage of post-1980s deregulatory policies, the pharmaceutical industry began to redirect its pipeline to new “lifestyle drugs” and its marketing to consumer advertising. Science and medicine journalists played key roles in whetting the public's appetite for medical news about sex by breathlessly covering each new discovery and treatment.

In the 1980s and 1990s, urologists created organizations, journals, and “sexual health clinics” that focused on men's erection problems. In 1992, a US National Institutes of Health consensus conference on “impotence” legitimized this work. Its outcome was a 34-page document that mentioned factors involved in etiology, maintenance, and treatment such as culture, partners, and sexual techniques, but, for the most part, it reified “erection” as the essence of men's sexuality, and called for new treatments and vastly expanded research into physiological details and treatments [ 8]. The creation of “erectile dysfunction” as a serious, prevalent, and treatable medical disorder was firmly in place by the time Viagra was launched in 1998 with an unprecedented global public-relations campaign, as Joel Lexchin describes in this issue of *PLoS Medicine* [ 9].

## Creating FSD

Although journalists began calling for a “female Viagra” only days after the March 1998 US Food and Drug Administration (FDA) approval of Viagra (examples of journalists’ calling for a “pink Viagra” are collected on http://www.fsd-alert.org/press.html), it was far from clear what medical condition Viagra was supposed to treat in women. Urologists had used the term “female sexual dysfunction” as early as 1997, referring to aspects of genital pathophysiology that might be akin to erectile dysfunction. Figure 1 offers a timeline of events shaping the creation and promotion of FSD, from 1997 to the present.

**Figure 1:**
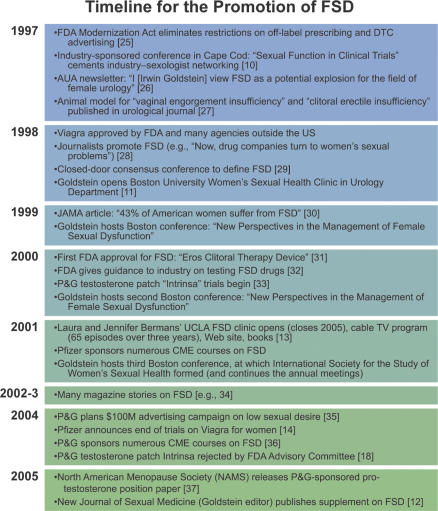
Timeline for the Promotion of FSD from 1997 to Present AUA, American Urological Association; CME, Continuing Medical Education; JAMA, Journal of the American Medical Association (Figure: Rusty Howson)

A May 1997 Cape Cod conference, “Sexual Function Assessment in Clinical Trials,” which was sponsored by pharmaceutical companies, was a watershed moment in the FSD story [ 10]. These companies bypassed existing sexology organizations and their annual conferences to convene an invitation-only industry–sexologist get-together. Papers and discussion were published in a special supplement to the *International Journal of Impotence Research* [ 10]. Significantly, the introduction stated:

*“In the area of female sexual dysfunction, there is widespread lack of agreement about the definition of sexual dysfunction, its pathophysiology or clinical manifestations, and the optimal approach for research or clinical assessment (p. S1).”*

Definitional issues have plagued the FSD literature ever since, despite repeated industry-supported attempts to draw a bright line between healthy sexual function and medical disorder. The quest for a valid and reliable FSD assessment instrument has become a small growth industry in and of itself.

For the first few years, the key players in the medicalization of women's sexual problems were a small group of urologists who capitalized on their relationships with industry and recruited many sex researchers and therapists as allies. Irwin Goldstein of Boston University, an active erectile dysfunction researcher, opened the first Women's Sexual Health clinic in 1998 [ 11]. He convened the first conference on female sexual function (called “New Perspectives in the Management of Female Sexual Dysfunction”) in October 1999 in Boston. Goldstein is the editor of a journal that launched in 2004—the *Journal of Sexual Medicine* ( http://jsm.issir.org)—which has already published an industry-supported supplement on FSD [ 12].

Jennifer Berman, Goldstein's urology trainee at Boston University, together with her sister, sex educator Laura Berman, became the female face of FSD, opening a clinic at University of California Los Angeles (UCLA) in 2001, and continuing to popularize FSD and off-label drug treatments on their television program, Web site, and books; in appearances on the television show “Oprah”; and in innumerable women's magazines [ 13]. The UCLA clinic was closed in 2005, as both Jennifer (in Los Angeles, California) and Laura (now in Chicago, Illinois) opened fee-for-service women's sexual-health centers that offered medical assessments and treatments plus spa and yoga services [ 13]. Laura will also have her own reality TV sex-advice show later in 2006 ( http://www.sho.com/site/announcements/051005sexual.do). One clear future angle to the FSD story will be its intersection with the new “holistic” and “boutique” (specialized, retainer, or cash-paying) medical trends as well as with drug-friendly celebrity experts.

Pfizer, the world's largest pharmaceutical company, was the main promoter of FSD from 1997 to 2004, when its quest to have Viagra approved to treat “female sexual arousal disorder” ended because of consistently poor clinical-trial results. In its public statement, Pfizer said that that several large-scale, placebo-controlled studies including about 3,000 women with female sexual arousal disorder showed inconclusive results on the efficacy of the drug [ 14]. Commenting on these trial results on Viagra, John Bancroft, director of the Kinsey Institute, told the *BMJ*: “The recent history of the study of female sexual dysfunction is a classic example of starting with some preconceived, and non-evidence based diagnostic categorisation for women's sexual dysfunctions, based on the male model, and then requiring further research to be based on that structure. Increasingly it is becoming evident that women's sexual problems are not usefully conceptualised in that way” [ 14]. Nevertheless, Viagra (and the idea that it *must* work for women) has been so successfully branded that it continues to be prescribed off-label for women [ 15].

Next in line with a potential drug for FSD has been Procter & Gamble (P&G), the multibillion-dollar soap, shampoo, and snack company that makes only five prescription drugs [ 16]. P&G's 2004 annual report states that its drug risedronate (Actonel, approved in 1998 for Paget disease and in 2000 for osteoporosis), “became a billion-dollar brand faster than any other brand in P&G history” [ 17]. Perhaps encouraged by this success in selling medicine to women, P&G had begun investing heavily in a testosterone patch (brand name Intrinsa) to treat “hypoactive sexual desire disorder.” The unnoticed shift in 2004 in FSD identity and promotion from female sexual arousal disorder to hypoactive sexual desire disorder is another hallmark moment in the FSD story, illustrating how the effort to match up some drug with FSD moved freely among symptoms and labels. P&G's trials with Intrinsa got many gynecologists and their organizations heavily involved in the new sexual pharma–medicine for the first time. Unfortunately for the drug company, an FDA advisory panel voted unanimously not to approve Intrinsa, saying that P&G had not provided sufficient long-term safety data and questioning the clinical significance of the Intrinsa trials [ 18]. However, testosterone researcher Jan Shifren estimates that one-fifth of all the prescriptions of testosterone products approved for men are actually written (off-label) for women [ 19].

By 2006, FSD has become a medical and media reality, despite the obvious ongoing difficulties in defining the condition and in getting a drug approved. Disease mongering has led to the successful “branding” of FSD.

## Activist Response

In 1999, I became concerned that the imminent inaugural Boston conference on FSD would represent only the reductionist view of women's sexual problems and would likely ignore the fundamental political and interpersonal reality of women's sexual lives. I had been employed as a research and clinical psychologist in urology departments from 1983 to 1996, and I worried that the mechanistic view of sexuality I had seen applied to men's sexual function would just be transposed to women. Viagra had just been approved, I knew about the Cape Cod conference, and I feared that urologists (with financial backing from Pfizer) would use a conference on FSD to promote Viagra for women.

Although I had no experience in organizing, I felt I had to take steps to make sure a space was created for diverse (i.e., not just medical) opinions about women's sexual problems. I submitted a critical essay about the new FSD to a Boston feminist newsletter [ 20], and, with Carol Tavris, I wrote an oped for the *Los Angeles Times* [ 21]. Through Internet communication, I invited feminist critics of medicalization to meet with me in Boston and take some action at the FSD conference. Figure 2 offers a timeline, beginning in 1999, of the activism that came to be called the “Campaign for a New View of women's sexual problems” [ 22].

**Figure 2:**
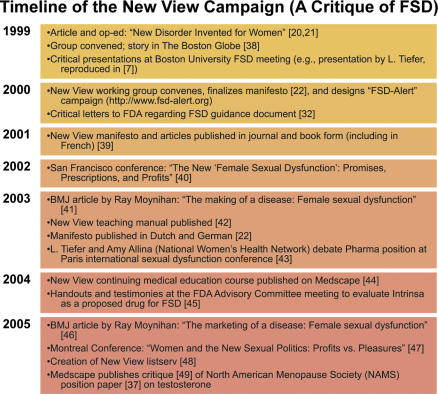
Timeline of Events Beginning in 1999 of Activism, Which Came to Be Called the “Campaign for a New View of Women's Sexual Problems” (Figure: Rusty Howson)

The pharmaceutical industry has taken an aggressive interest in sex.

The campaign and its challenge to FSD disease mongering have had two crucial components [ 23]. The first, a theoretical critique of the medical model of sexual problems, was developed in the New View Manifesto, books, articles, and lectures. The manifesto, now available in several languages [ 22], was authored by a group of feminist academics, activists, and clinicians calling themselves “The Working Group on a New View of Women's Sexual Problems.” The second component of the campaign is pharma–watchdog activism, consisting of media interviews, conferences, FDA and professional presentations, and a Web site ( http://fsd-alert.org).

The New View Manifesto focuses on weaknesses of the prevailing sexual dysfunction classification and medical model. It promotes a politically sensitive social-constructionist perspective and recommends abandoning the effort to define “normal” sexual function. It offers an alternative classification system of causes for sexual problems rooted in society, relationships, psychology, and disease. The activism challenges claims made for each emerging FSD drug in terms based on recurring biases in clinical trials, dangers of off-label promotion, researchers’ conflicts of interest, and neglect of nonmedical theory and research on sexuality.

## Conclusion

Sexual life and its pleasures, problems, and satisfactions are subject to changing demands and expectations. Recently, the pharmaceutical industry has taken an aggressive interest in sex, using public relations, direct-to-consumer advertising, promotion of off-label prescribing, and other tactics to create a sense of widespread sexual inadequacy and interest in drug treatments.

The public finds medicalization attractive because the notion of simple but scientific solutions fits in with a general cultural overinvestment in biological explanations and interventions, and promises to bypass sexual embarrassment, ignorance, and anxiety. This wish will inevitably end in stories of personal disappointment, but media promotion, advertising hyperbole, and an active pipeline will create continuing hope for the next new drug along with a neglect of other models of sex and ways to deal with sexual discontent.

The New View Campaign to challenge the disease mongering of FSD can be seen as part of a widespread new arena of public-health advocacy that deals with corporate practices that affect health, such as those in the tobacco, automobile, and food industries [ 24]. Activism on behalf of women's sexuality leads also to coalition with sexual-rights, sex-education, and reproductive-rights organizations. It has taken the work of many public-spirited people and organizations to shed the necessary light on FSD disease mongering. But the difficulties the industry and its experts continue to have in nailing down FSD testify to some small success on our part.

**The Major Disease-Mongering Tactics Identified by Lynn Payer [ 1]**

1. “Taking a normal function and implying that there's something wrong with it and it should be treated” (p. 88)
2. “Imputing suffering that isn't necessarily there” (p. 89)
3. “Defining as large a proportion of the population as possible as suffering from the ‘disease’” (p. 89)
4. “Defining a [condition] as a deficiency disease or disease of hormonal imbalance” (p. 93)
5. “Getting the right spin doctors” (p. 93)
6. “Framing the issues in a particular way” (p. 94)
7. “Selective use of statistics to exaggerate the benefits of treatment” (p. 95)
8. “Using the wrong end point” (p. 96)
9. “Promoting technology as risk-free magic” (p. 96)
10. “Taking a common symptom that could mean anything and making it sound as if it is a sign of a serious disease” (p. 98)

## Abbreviations

FDA: US Food and Drug Administration
FSD: female sexual dysfunction
P&G: Procter & Gamble
UCLA: University of California Los Angeles
